# High-risk patients for septic shock after percutaneous nephrolithotomy

**DOI:** 10.1590/S1677-5538.IBJU.2024.0154

**Published:** 2024-07-20

**Authors:** Alexandre Danilovic, Lucas Piraciaba Cassiano Dias, Fabio Cesar Miranda Torricelli, Giovanni Scala Marchini, Carlos Batagello, Fabio Carvalho Vicentini, William C. Nahas, Eduardo Mazzucchi

**Affiliations:** 1 Faculdade de Medicina da Universidade de São Paulo Hospital das Clínicas Departamento de Urologia São Paulo SP Brasil Departamento de Urologia, Hospital das Clínicas, Faculdade de Medicina da Universidade de São Paulo, São Paulo, SP, Brasil; 2 Hospital das Clínicas da Faculdade de Medicina da Universidade de São Paulo São Paulo SP Brasil Disciplina de Urologia do Hospital das Clínicas da Faculdade de Medicina da Universidade de São Paulo, São Paulo, SP, Brasil

**Keywords:** Kidney Calculi, Nephrolithotomy, Percutaneous, Postoperative Complications

## Abstract

**Purpose:**

to identify risk factors for urinary septic shock in patients who underwent percutaneous nephrolithotomy (PCNL).

**Materials and Methods:**

Data from PCNL procedures performed between January 2009 and February 2020 were retrospectively analyzed. The study included all patients over 18 years old with kidney stones larger than 15 mm who underwent PCNL. Patients who underwent mini-PCNL or combined surgeries, such as ureteroscopy or bilateral procedures, were not included in the study. Logistic regression was conducted to determine the risk factors for urinary septic shock within 30 days post-operation in patients who underwent PCNL.

**Results:**

Urinary septic shock was observed in 8 out of the 1,424 patients analyzed (0.56%). The presence of comorbidities, evaluated using the Charlson Comorbidity Index (CCI) (OR 1.46 [CI 95% 1.15-1.86], p=0.01), larger stones (41.0 mm [IQR 30.0-47.5 mm] vs. 24.0 mm [IQR 17.0-35.0 mm], OR 1.03 [CI 95% 1.01-1.06], p=0.04), and a positive preoperative urine culture (OR 8.53 [CI 95% 1.71-42.45], p <0.01) were shown to significantly increase the risk of postoperative urinary septic shock. Patients with a CCI > 2, larger stones (≥ 35 mm), and a positive preoperative urine culture were at even higher risk of urinary septic shock (OR 15.40 [CI 95% 1.77-134.21], p=0.01).

**Conclusion:**

Patients with larger stones, positive preoperative urine culture, and a higher CCI are at risk for urinary septic shock after PCNL. These findings are of utmost importance for optimizing the perioperative care of these patients to prevent life-threatening complications.

## INTRODUCTION

Percutaneous nephrolithotomy (PCNL) is the first line therapy for large kidney stones (1). Despite the reduction in complications associated with PCNL due to technological improvements, postoperative infectious complications remain a significant challenge (2-4). Approximately 10% of the patients experience transient postoperative fever, while 0.3-0.5% develop urinary sepsis after PCNL (5, 6). Although rare, sepsis is a dramatic complication and constitutes the primary cause of mortality among patients undergoing PCNL (7).

The literature lacks a clear consensus on defining high-risk patients for postoperative infectious complications of PCNL. Most previous studies use postoperative fever as their primary endpoint, and the changing definitions of the terms "sepsis" and "septic shock" in recent years have led to different outcomes (8). A prospective study demonstrated that postoperative fever is associated with *diabetes mellitus*, positive urine culture (particularly Gram-negative pathogen), staghorn calculus, and the preoperative use of a nephrostomy tube (6). Other authors have demonstrated that the need for blood transfusion, larger stone size and prolonged operative time were risk factors for post-PCNL sepsis. Possible predictors, such as positioning in PCNL, tract size, obesity, and a solitary kidney did not impact infectious rates in most of the previous studies (9-13). As a result, there are no specific recommendations for antibiotic prophylaxis due to a lack of sufficient data (14-16). A recent meta-analysis demonstrated a decrease in the risk of sepsis in the postoperative period of PCNL with the use of oral antibiotic prophylaxis for one week before surgery, regardless of the patient risk factors (15). In order to optimize the use of perioperative antibiotics for PCNL, we need more studies to better understand the risk factors for severe infectious complications of PCNL such as septic shock. Currently, EAU Guidelines states that the available evidence for prevention of infection following percutaneous stone removal remains limited (1).

Our hypothesis is that identifying high infectious risk patients for PCNL would facilitate the establishment of specific recommendations for antibiotic prophylaxis and the reduction of infectious complications in these patients. The primary objective of this study is to evaluate predictors associated with the progression to postoperative PCNL sepsis shock resulting from urinary tract infection (UTI).

## MATERIALS AND METHODS

Data from PCNL procedures performed between January 2009 and February 2020 at a single specialized center were retrospectively analyzed. The study included all patients over 18 years old with kidney stones larger than 15 mm who underwent PCNL. We did not restrict the inclusion of multiple events for a single patient. Patients who underwent mini-PCNL or combined surgeries, such as ureteroscopy or bilateral procedures, were not included in the study. The institutional ethics committee approved this study (IRB approval number 75906623.0.0000.0068).

Personal clinical features, such as age, gender, body mass index (BMI), and comorbidities, were evaluated. These factors were analyzed using the Charlson Comorbidity Score (CCI) and the *American Society of Anesthesiologists* score (ASA) (17, 18). Preoperative urinary drainage using a ureteral catheter or a nephrostomy tube and whether the event was indicated as a second-look surgery were also evaluated. Radiological characteristics of the stones and the urinary tract were assessed by preoperative non-contrast computed tomography (NCCT) (19). These features included the largest diameter of the addressed stones, the presence of hydronephrosis, and the analysis of the *Guy's Stone Score* (20).

Patients underwent PCNL in either the prone position or one of the supine position variations (complete supine, Galdakao modified Valdivia, or Barts flank-free position), which are commonly employed at our Institution, selected according to the surgeon's preference. All patients received a third-generation cephalosporin during the induction of anesthesia, except for those with a positive urine culture who received antibiotics guided by the urine culture results. A negative urine culture was obtained before the procedure. Following the general anesthesia, we initiated the procedure with a cystoscopy to place a 6 French (Fr) ureteral catheter up to the side of the addressed stone. After proper positioning, the calyceal punctures were guided by a retrograde pyelogram, ultrasound, or a combination of both techniques. The procedure was interrupted if pyuria was observed by the surgeon, and a nephrostomy tube was then inserted. Subsequently, the urinary tract was dilated using plastic dilators (Amplatz dilators), and nephroscopy was performed using a 26-F nephroscope (Karl Storz, Tuttlingen, Germany). Stone fragmentation was achieved using an ultrasonic lithotripter (Swiss Lithoclast Master, EMS, Switzerland). At the conclusion of the procedure, either a ureteral stent or a nephrostomy tube was placed based on the surgeon's preference. Stone-free status was routinely assessed by NCCT performed on the first postoperative day (POD) (21, 22).

Additionally, we conducted a microbiological analysis, which involved a urine culture collected on the day before the procedure, an analysis of the composition of the calculus, and the culture of the calculus itself. Regarding perioperative data, we analyzed the operative time and hematimetric variation.

We employed a dual-check approach to define sepsis. We combined the diagnosis provided during the event with a retrospective diagnosis based on the updated Sepsis-3 criteria: sepsis as organ dysfunction caused by the response to infection, utilizing the diagnosis of infection and a *Sequential Organ Failure Assessment (SOFA)* score ≥ 2. Septic shock definition was a circulatory dysfunction identified by a vasopressor requirement to maintain a mean arterial pressure of 65 mmHg and serum lactate level >2 mmol/L (>18 mg/dL) (23). Additionally, we checked the data of all patients who required intensive care in the postoperative period following PCNL.

### Statistical Analysis

Measures of central tendency and dispersion, as well as absolute and relative frequencies, were calculated for participant characterization and the analysis of clinical and sociodemographic characteristics of participants, stratified by the presence or absence of urinary septic shock.

Due to the dichotomous nature of the dependent variable, we employed logistic regression for bivariate analyses. We adopted a significance level of α < 0.05 and a 95% confidence interval (CI 95%). The Stata/SE software version 12 (StataCorp LLC®, United States) was utilized for data analysis.

## RESULTS

Among the analysis of the 1,424 surgical procedures, we observed a predominance of women (63.3%), with a median age of 47 years and a BMI of 27.8 kg/m² (Table-1). The primary outcome of postoperative urinary septic shock occurred in 8 of the 1,424 patients (0.56%). We recorded two deaths due to septic shock, resulting in a lethality rate of 0.14% in our study. One patient died on the 7 POD and the other on the 12 POD due to organic dysfunction caused by urinary sepsis.

**Table 1:** Clinical features of 1424 patients included in the study.

Features			(n) (%) or Median [IQR]
Female			903 (63.4)
Age			47.0 [36.0-57.0]
BMI			27.8 [24.4-31.9]
**ASA score**			
		1	459 (32.3)
		2	836 (58.8)
		3	126 (8.8)
		4	2 (0.1)
**Charlson Comorbidity Index**			
		0	664 (46.7)
		1	336 (23.6)
		2	220 (15.5)
		3	122 (8.6)
		4	43 (3.0)
		5	18 (1.3)
		6	12 (0.8)
		7	3 (0.2)
		8	1 (0.1)
		9	1 (0.1)
		10	2 (0.1)
		13	1 (0.1)
Stone diameter (mm)			24.0 [17.0-35.0]
Guys's score			
		1	67 (4.7)
		2	426 (30.0)
		3	657 (46.3)
		4	268 (18.9)
Hydronephrosis			906 (64.8)
Preoperative ureteral stent			287 (20.2)
Preoperative urinary drainage			314 (22.1)
Second-look surgery			96 (6.8)
**Urine culture**			
	**Positive**		368 (26.1)
		Escherichia coli	138 (9.8)
		Proteus mirabilis	55 (3.9)
		Group B Streptococcus	36 (2.6)
		Coagulase-negative Staphylococcus	30 (2.1)
		Klebsiella pneumoniae	28 (2.0)
		Pseudomonas aeruginosa	16 (1.1)
	Staphylococcus aureus		10 (0.7)
	Other bacteria and fungi		55 (3.9)
Negative			1039 (73.9)
**Stone culture**			
	**Positive**		300 (36.1)
		Coagulase-negative Staphylococcus	79 (9.5)
		Escherichia coli	66 (8.0)
		Proteus spp	39 (4.7)
		Enterococcus spp	29 (3.5)
		Klebsiella pneumoniae	17 (2.0)
		Pseudomonas spp	17 (2.0)
		Staphylococcus aureus	10 (1.2)
		Streptococcus viridans	8 (1.0)
	Other bacteria and fungi		35 (4.2)
Negative			530 (63.9)
**Stone composition**			
Struvite			630 (50.6)
Calcium oxalate			273 (21.9)
Mixed calcium stones (oxalate, phosphate, carbonate, uric acid)			205 (16.4)
Calcium oxalate + calcium phosphate			83 (6.7)
Uric acid			41 (3.3)
Cystine			14 (1.1)
Stone free procedures			684 (48.3)
Operative time, minutes			120 [90-160]
Hematocrit drop (%)			4.3 [1.9 -7.2]

IQR = interquartile range; BMI = Body Mass Index; ASA = American Society of Anesthesiologists; mm = milimeter

Hemorrhagic shock was the cause of admission to the intensive care unit (ICU) in eight patients, with two deaths in this group. An additional eight patients were admitted to the ICU for various reasons, including renal insufficiency, iatrogenic colon injury, drug-induced hepatitis, adrenal insufficiency, and acute cardiac decompensation, resulting in one death due to acute myocardial infarction.

The presence of comorbidities, as assessed by the CCI, demonstrated a significant risk of postoperative urinary septic shock (OR 1.46 [CI 95% 1.15-1.86], p=0.01). Patients who developed urinary septic shock had a median CCI score of 2 [IQR 0-5] vs. 1 [IQR 0-2] (Table-2). Larger stones also showed a higher risk of progressing to urinary septic shock (OR 1.03 [CI 95% 1.01-1.06], p=0.04). The median stone size for patients who developed urinary septic shock was 41.0 mm [IQR 30.0-47.5 mm] vs. 24.0 mm [IQR 17.0-35.0 mm] in other patients. We used the highest interquartile range of the patients who did not develop urinary septic shock to analyze stone size as categorical data. Stones ≥ 35.0 mm demonstrated a higher risk of progressing to urinary septic shock when compared to smaller stones (OR 4.51 [CI 95% 1.08-18.94], p=0.04). A positive preoperative urine culture was associated with a higher chance of urinary septic shock (OR 8.53 [CI 95% 1.71-42.45], p <0.01). Patients with CCI > 2, larger stones (≥ 35 mm), and a positive preoperative urine culture were at even higher risk of urinary septic shock (OR 15.40 [CI 95% 1.77-134.21], p=0.01).

**Table 2:** Analysis of predictors for urinary septic shock after PCNL.

Features		Total (N=1,424) (%)	Urinary septic shock (N = 8) (%)	No urinary septic shock (N = 1,416) (%)	p value	*Odds ratio [CI 95%]* *
**Gender**						
	Female	903 (63.4)	5 (62.5)	898 (63.4)	0.96	
Age		47.0 [36.0-57.0]	53.0 [36.5-65.5]	47.0 [36.0-57.0]	0.22	
BMI		27.8 [24.4-31.9]	26.4 [23.4-28.5]	27.8 [24.4-32.0]	0.34	
**ASA score**						
	1	459 (32.3)	2 (25.0)	457 (32.3)	0.08	
	2	836 (58.8)	3 (37.5)	833 (58.9)		
	3	126 (8.8)	3 (37.5)	123 (8.7)		
	4	2 (0.1)	0 (0.0)	2 (0.1)		
	CCI	1 [0-2]	2 [0-5]	1 [0-2]	0.01	1.46 [1.15-1.86]
Diabetes		136 (9.6)	2 (25.0)	134 (9.5)	0.20	
**Stone diameter (mm)**		24.0 [17.0-35.0]	41.0 [30.0-47.5]	24.0 [17.0-35.0]	0.04	1.03 [1.01-1.06]
Stone diameter (mm)						
	< 35mm	1032 (72.8)	3 (37.5)	1029 (73.0)	0.04	4.50 [1.08-18.93]
	≥ 35mm	386 (27.2)	5 (62.5)	381 (27.0)		
**Guy's score**						
	1	67 (4.7)	0 (0.0)	67 (4.7)	0.90	
	2	426 (30.0)	2 (25.0)	424 (30.1)		
	3	657 (46.3)	4 (50.0)	653 (46.3)		
	4	268 (18.9)	2 (25.0)	266 (18.9)		
Hydronephrosis		906 (64.8)	6 (75.0)	900 (64.7)	0.53	
Previous urinary drainage		314 (22.1)	3 (37.5)	311 (22.0)	0.32	
Second-look surgery		101 (7.1)	0 (0.0)	101 (7.1)	---	
**Urine culture**						
	Positive	370 (26.3)	6 (75.0)	364 (26.0)	<0.01	8.53 [1.71-42.45]
	*E. coli*	138 (9.8)	5 (62.5)	133 (9.5)	<0.01	19.45 [3.73-101.27]
	Other bacteria	232 (16.5)	1 (12.5)	231 (16.5)		2.24 [0.20-24.81]
	Negative	1037 (73.7)	2 (25.0)	1035 (74.0)		1
**Positive urine culture + Stone ≥ 35 mm**		116 (8.3)	4 (50.0)	112 (8.0)	<0.01	11.46 [2.83-46.42]
**CCI > 2 + Positive urine culture + Stone ≥ 35 mm**		14 (1.0)	1 (12.5)	13 (0.9)	0.01	15.40 [1.77-134.21]
**Stone culture**						
	*E. coli*	66 (7.9)	2 (33.3)	64 (7.8)	0.36	
	Other bacteria	234 (28.2)	0 (0.0)	234 (28.4)		
	Negative	530 (63.9)	4 (66.7)	526 (63.8)		
**Stone composition**						
Struvite		630 (50.6)	5 (62.5)	625 (50.5)	0.32	
Calcium oxalate		273 (21.9)	1 (12.5)	272 (22.0)		
Mixed calcium stones (oxalate, phosphate, carbonate, uric acid)		205 (16.4)	0 (0.0)	205 (16.6)		
Calcium oxalate + calcium phosphate		83 (6.7)	1 (12.5)	82 (6.6)		
Uric acid		41 (3.3)	1 (12.5)	40 (3.2)		
Cystine		14 (1.1)	0 (0.0)	14 (1.1)		
Stone free rate		684 (48.3)	3 (37.5)	681 (48.4)	0.54	
Operative time, minutes		120 [90-160]	125 [120-145]	120 [90-160]	0.99	
Prone position		675 (47.4)	6 (75.0)	669 (47.3)	0.12	
Hematocrit drop (%)		4.3 [1.9 -7.2]	5.5 [2.5-6.5]	4.3 [1.9 -7.2]	0.91	

* Calculated only for those that showed statistical significance.

BMI = Body Mass Index; ASA = American Society of Anestesiologists; CCI = Charlson Comorbidity Index; mm = milimeter

*Escherichia coli* was the most frequent agent causing urinary septic shock (OR 19.45 CI [3.73-101.27], p <0.01), as it was present in five out of eight patients who experienced the primary outcome. Among the other three individuals, we observed one patient with a preoperative urine culture isolating *K. pneumoniae* and two patients with negative urine cultures, both of whom had a history of obstructive pyelonephritis.

The culture of the stones tested positive in 300 individuals (36.0%, n=830). Coagulase-negative *Staphylococcus* was isolated in 79 stone cultures (26.3% of the agents) and *E. coli* in 66 cases (22.0%). Among other isolated bacteria, there were 39 cases of *Proteus spp*, 29 cases of *Enterococcus spp*, 17 cases of *Klebsiella pneumoniae*, 17 cases of *Pseudomonas spp*, 10 cases of *Staphylococcus aureus*, and eight cases of *Streptococcus viridans.* Two individuals with positive cultures of the stone for E. coli developed urinary septic shock in the postoperative period, constituting one-third of patients from whom calculus culture was obtained, as is the only agent isolated in this group of patients, as depicted in Table-2.

A total of 1,246 events were evaluated with an analysis of calculus composition, resulting in 630 struvite stones (50.6%), 273 calcium oxalate stones (21.9%), 41 uric acid calculi (3.3%), 38 calcium oxalate and phosphate calculi (6.17%), 20 cystine calculi (1.1%), and the remaining composed of mixed stones containing calcium oxalate, calcium phosphate, calcium carbonate and uric acid.

## DISCUSSION

This study demonstrates that a higher CCI, larger renal stones, and a positive preoperative urine culture are risk factors for urinary septic shock in patients undergoing PCNL. Furthermore, for the first time we demonstrated a 15-fold increase in the risk of urinary septic shock with the association between these factors, regardless of operative time or patient position during PCNL. Preoperative knowledge of these new findings may assist in identifying high-infectious risk patients for this life-threatening complication to optimize perioperative management for such individuals.

Our study has shown that a higher CCI is a risk factor for developing urinary septic shock. Our results confirm the greater vulnerability following the manipulation of stones in these patients, which may be associated with their chronic state of immunosuppression. Gutierrez *et al.* also studied the relationship between comorbidities and post-PNCL infectious complications, demonstrating that *diabetes mellitus* was a risk factor for postoperative fever (OR = 1.38, [CI 95% 1.05-1.81]) (6). However, it is important to distinguish the differences between the analyzed endpoints. Most postoperative fevers are transient and benign situations in which patients do not develop the life-threatening condition of septic shock. Additionally, there are some risks of bias in the postoperative fever outcome due to the routine use of antipyretic drugs in the postoperative period and the different temperature thresholds used to define fever among distinct studies.

Larger kidney stones presented a greater risk of septic shock in our study. Large-sized stones may be associated with the increased release of endotoxins and bacteria into the bloodstream (24, 25). This observation may be connected to the formation of infectious calculi. These stones tend to be larger, demanding procedures that last longer. However, our study failed to demonstrate that prolonged operative time was a risk factor for urinary septic shock. Wang et al., in a retrospective study involving 420 patients, found that operative time > 90 min was a significant risk factor for septic shock (26). Recently, Bansal et al. demonstrated in their retrospective study (N=580) that, in addition to prolonged operative time and the need for blood transfusions, the presence of stones >25mm was a risk factor for post-PCNL sepsis (27). There is no consensus about the threshold stone size that implies a higher risk of infectious complications. Many studies assessed stone size with a cutoff of 20 or 25mm. However, this dimension is usually associated with the stone size for PCNL indication, making it difficult to distinguish between risk groups. Therefore, our finding of a cutoff of 35 mm as a risk factor for urinary septic shock helps to identify high infectious risk patients more effectively.

Bacteriuria diagnosed in a preoperative urine culture is a risk factor for urinary septic shock. Moreover, the isolation of E. coli and gram-negative bacilli (GNB) also demonstrated a statistically significant risk association. Nowadays, there is evidence that urine culture collected directly from the renal pelvis is considered more indicative of the causative organism of sepsis (28). Coagulase-negative Staphylococcus was the most frequently isolated agent from the culture of the stones. However, it was not associated with any cases of urinary septic shock, in contrast to the isolation of E. coli, which was associated with two patients experiencing this outcome. These findings may indicate contamination rather than the development of an infection.

Previous studies also demonstrated that preoperative infected urine is a risk factor for infection after PCNL. Xun *et al*. conducted a retrospective study with 745 patients, in which positive urine culture (OR 3.24, p=0.025) was associated with sepsis (29). A meta-analysis published by Lai et al., based on 12 prospective studies (n=1348), concluded that positive preoperative urine culture (OR 2.14, p=0.026), positive intraoperative renal pelvis culture (OR 8.27, p=0.0001) and positive stone culture (OR 5.68, p=0,0001) were predictive factors for postoperative infection (30). The Clinical Research Office of the Endourological Society (CROES) collected prospective data from 5,803 patients treated with PCNL. The authors demonstrated that postoperative fever (≥ 38.5°C) is associated with positive urine culture (especially GNB), *diabetes mellitus*, the presence of a staghorn calculus, and a preoperative nephrostomy tube (5). In our study, neither hydronephrosis nor previous urinary drainage (ureteral stent or nephrostomy) demonstrated an association with urinary septic shock.

Antibiotic prophylaxis may be associated with a reduction in postoperative infectious outcomes. A recent meta-analysis demonstrated a decrease in the risk of sepsis in the postoperative period of PCNL with the use of oral antibiotic prophylaxis for one week before surgery, regardless of the patient risk factors (15). However, this is still a controversial topic in the literature, and the lack of identification of high-risk patients for postoperative infectious complications limits the optimal use of antibiotics in patients who would benefit the most from treatment.

Our study has some limitations. This is a retrospective study from a single institution. Nevertheless, the study was based on a large, prospectively filled electronic medical records database. PCNL was performed by different surgeons in this retrospective study. Variability in surgical proficiency among different surgeons may impact the results of the study outcomes, as in real life different surgeons may have different outcomes of the same procedure. Additionally, the rarity of the primary endpoint prevents us from prospectively assessing the risk factors for urinary septic shock in patients undergoing PCNL.

## CONCLUSION

Patients with stones larger than 35mm, positive preoperative urine culture, and a higher CCI are at higher risk for urinary septic shock after PCNL. These findings are of utmost importance to optimize the perioperative care of these patients to prevent life-threatening complications.

## References

[1] Türk C, Petřík A, Sarica K, Seitz C, Skolarikos A, Straub M (2016). EAU Guidelines on Interventional Treatment for Urolithiasis. Eur Urol.

[2] Qin P, Zhang D, Huang T, Fang L, Cheng Y (2022). Comparison of mini percutaneous nephrolithotomy and standard percutaneous nephrolithotomy for renal stones >2cm: a systematic review and meta-analysis. Int Braz J Urol.

[3] Danilovic A, Torricelli FCM, Marchini GS, Batagello C, Vicentini FC, Traxer O (2021). Residual Stone Fragments After Percutaneous Nephrolithotomy: Shockwave Lithotripsy vs Retrograde Intrarenal Surgery. J Endourol.

[4] Kumar S, Keshavamurthy R, Karthikeyan VS, Mallya A (2017). Complications after prone PCNL in pediatric, adult and geriatric patients - a single center experience over 7 years. Int Braz J Urol.

[5] de la Rosette J, Assimos D, Desai M, Gutierrez J, Lingeman J, Scarpa R (2011). The Clinical Research Office of the Endourological Society Percutaneous Nephrolithotomy Global Study: indications, complications, and outcomes in 5803 patients. J Endourol.

[6] Gutierrez J, Smith A, Geavlete P, Shah H, Kural AR, de Sio M (2013). Urinary tract infections and post-operative fever in percutaneous nephrolithotomy. World J Urol.

[7] Whitehurst L, Jones P, Somani BK (2019). Mortality from kidney stone disease (KSD) as reported in the literature over the last two decades: a systematic review. World J Urol.

[8] Kreydin EI, Eisner BH (2013). Risk factors for sepsis after percutaneous renal stone surgery. Nat Rev Urol.

[9] Isoglu CS, Suelozgen T, Boyacioglu H, Koc G (2017). Effects of body mass index on the outcomes of percutaneous nephrolithotomy. Int Braz J Urol.

[10] Torricelli FC, Padovani GP, Marchini GS, Vicentini FC, Danilovic A, Reis ST (2015). Percutaneous nephrolithotomy in patients with solitary kidney: a critical outcome analysis. Int Braz J Urol.

[11] Ozdemir H, Erbin A, Sahan M, Savun M, Cubuk A, Yazici O (2019). Comparison of supine and prone miniaturized percutaneous nephrolithotomy in the treatment of lower pole, middle pole and renal pelvic stones: A matched pair analysis. Int Braz J Urol.

[12] Perrella R, Vicentini FC, Paro ED, Torricelli FCM, Marchini GS, Danilovic A (2022). Supine versus Prone Percutaneous Nephrolithotomy for Complex Stones: A Multicenter Randomized Controlled Trial. J Urol.

[13] Danilovic A, Torricelli FCM, Marchini GS, Batagello C, Vicentini FC, Traxer O (2021). Does previous standard percutaneous nephrolithotomy impair retrograde intrarenal surgery outcomes?. Int Braz J Urol.

[14] Geraghty RM, Davis NF, Tzelves L, Lombardo R, Yuan C, Thomas K (2023). Best Practice in Interventional Management of Urolithiasis: An Update from the European Association of Urology Guidelines Panel for Urolithiasis 2022. Eur Urol Focus.

[15] Danilovic A, Talizin TB, Torricelli FCM, Marchini GS, Batagello C, Vicentini FC (2023). One week pre-operative oral antibiotics for percutaneous nephrolithotomy reduce risk of infection: a systematic review and meta-analysis. Int Braz J Urol.

[16] Torricelli FCM, Monga M (2020). Staghorn renal stones: what the urologist needs to know. Int Braz J Urol.

[17] Charlson ME, Pompei P, Ales KL, MacKenzie CR (1987). A new method of classifying prognostic comorbidity in longitudinal studies: development and validation. J Chronic Dis.

[18] Sankar A, Johnson SR, Beattie WS, Tait G, Wijeysundera DN (2014). Reliability of the American Society of Anesthesiologists physical status scale in clinical practice. Br J Anaesth.

[19] Danilovic A, Rocha BA, Marchini GS, Traxer O, Batagello C, Vicentini FC (2019). Computed tomography window affects kidney stones measurements. Int Braz J Urol.

[20] Thomas K, Smith NC, Hegarty N, Glass JM (2011). The Guy's stone score--grading the complexity of percutaneous nephrolithotomy procedures. Urology.

[21] Guglielmetti GB, Danilovic A, Torricelli FC, Coelho RF, Mazzucchi E, Srougi M (2013). Predicting calyceal access for percutaneous nephrolithotomy with computed tomography multiplanar reconstruction. Clinics (Sao Paulo).

[22] Danilovic A, Cavalanti A, Rocha BA, Traxer O, Torricelli FCM, Marchini GS (2018). Assessment of Residual Stone Fragments After Retrograde Intrarenal Surgery. J Endourol.

[23] Singer M, Deutschman CS, Seymour CW, Shankar-Hari M, Annane D, Bauer M (2016). The Third International Consensus Definitions for Sepsis and Septic Shock (Sepsis-3). JAMA.

[24] Michel MS, Trojan L, Rassweiler JJ (2007). Complications in percutaneous nephrolithotomy. Eur Urol.

[25] Mariappan P, Smith G, Bariol SV, Moussa SA, Tolley DA (2005). Stone and pelvic urine culture and sensitivity are better than bladder urine as predictors of urosepsis following percutaneous nephrolithotomy: a prospective clinical study. J Urol.

[26] Wang Y, Jiang F, Wang Y, Hou Y, Zhang H, Chen Q (2012). Post-percutaneous nephrolithotomy septic shock and severe hemorrhage: a study of risk factors. Urol Int.

[27] Bansal SS, Pawar PW, Sawant AS, Tamhankar AS, Patil SR, Kasat GV (2017). Predictive factors for fever and sepsis following percutaneous nephrolithotomy: A review of 580 patients. Urol Ann.

[28] Liu M, Cui Z, Zhu Z, Gao M, Chen J, Zeng F (2022). Development of a Nomogram Predicting the Infection Stones in Kidney for Better Clinical Management: A Retrospective Study. J Endourol.

[29] Xun Y, Yang Y, Yu X, Li C, Lu J, Wang S (2020). A preoperative nomogram for sepsis in percutaneous nephrolithotomy treating solitary, unilateral and proximal ureteral stones. PeerJ.

[30] Lai WS, Assimos D (2018). Factors Associated With Postoperative Infection After Percutaneous Nephrolithotomy. Rev Urol.

